# Cryo-electron microscopy-based drug design

**DOI:** 10.3389/fmolb.2024.1342179

**Published:** 2024-03-04

**Authors:** Ecenur Cebi, Joohyun Lee, Vinod Kumar Subramani, Nayeon Bak, Changsuk Oh, Kyeong Kyu Kim

**Affiliations:** Department of Precision Medicine, Sungkyunkwan University School of Medicine, Suwon, Republic of Korea

**Keywords:** structure-based drug design, cryo-electron microscopy, drug development, high-resolution, single particle analysis

## Abstract

Structure-based drug design (SBDD) has gained popularity owing to its ability to develop more potent drugs compared to conventional drug-discovery methods. The success of SBDD relies heavily on obtaining the three-dimensional structures of drug targets. X-ray crystallography is the primary method used for solving structures and aiding the SBDD workflow; however, it is not suitable for all targets. With the resolution revolution, enabling routine high-resolution reconstruction of structures, cryogenic electron microscopy (cryo-EM) has emerged as a promising alternative and has attracted increasing attention in SBDD. Cryo-EM offers various advantages over X-ray crystallography and can potentially replace X-ray crystallography in SBDD. To fully utilize cryo-EM in drug discovery, understanding the strengths and weaknesses of this technique and noting the key advancements in the field are crucial. This review provides an overview of the general workflow of cryo-EM in SBDD and highlights technical innovations that enable its application in drug design. Furthermore, the most recent achievements in the cryo-EM methodology for drug discovery are discussed, demonstrating the potential of this technique for advancing drug development. By understanding the capabilities and advancements of cryo-EM, researchers can leverage the benefits of designing more effective drugs. This review concludes with a discussion of the future perspectives of cryo-EM-based SBDD, emphasizing the role of this technique in driving innovations in drug discovery and development. The integration of cryo-EM into the drug design process holds great promise for accelerating the discovery of new and improved therapeutic agents to combat various diseases.

## 1 Introduction

**FIGURE 1:**
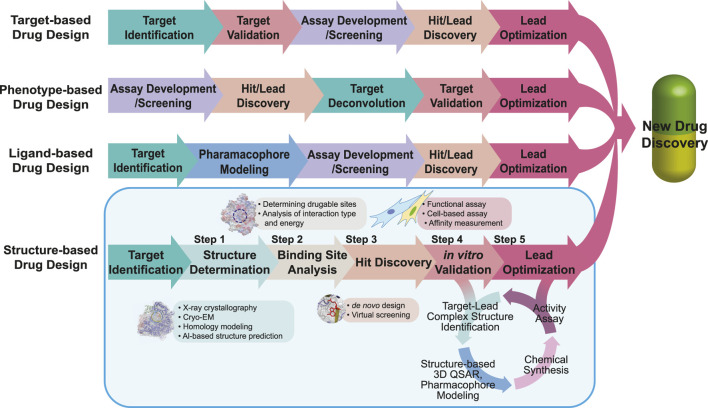
Workflow of drug design. The workflows of target-, phenotype-, ligand-, and structure-based drug designs are compared. The same color is used to describe the steps in the same category. For SBDD, the main focus of the review, we describe applicable tools in each step highlighted in the light blue box. In the SBDD section, the methods and tools applied in each step are listed in the boxes near each step.

The number of newly approved drugs has not notably increased recently, with an average of 49 drugs approved annually in the last 5 years and only 13 drugs approved in the second quarter of 2023 by the United States Food and Drug Administration (FDA) (Mullard, 2023; Urquhart, 2023). Moreover, newly approved drugs tend to focus on a limited range of diseases including oncological, neurological, and infectious diseases (Mullard, 2023). Approximately 40% of drug targets are G protein-coupled receptors (GPCRs), kinases, and ion channels, further narrowing the scope of potential drug targets (Santos et al., 2017). To address this limitation, the repertoire of drug targets should be expanded to cover a wider range of diseases including rare and genetic conditions (Smith et al., 2022). The reason for the limited number of new drug approvals lies in the extensive resources required for drug discovery, including time, expenses, interdisciplinary knowledge, and advanced technologies (Simoens and Huys, 2021; Van Norman, 2016; Wagner et al., 2018). Drug repositioning is one of the solutions for reducing risk factors and saving resources; however, further improvements are still required (Pushpakom et al., 2019). Drug discovery is a complex and risky process, with a high likelihood of failure, and overcoming these challenges and managing the risk of failure are essential for successful drug development (Sun et al., 2022). Several approaches have been applied to pursue low-risk and effective paths in drug discovery as following: 1) a target-based approach, which is screening chemicals on an *in vitro* system (e.g., the identified target molecules) (Terstappen et al., 2007; Croston, 2017); 2) a phenotype-based approach, which is screening chemicals on an *in vivo* system (e.g., cells, tissues, and animals with a reporter system or endogenous phenotype) (Moffat et al., 2017); 3) a ligand-based approach, using 3D structure-activity relationships (3D-QSAR) and pharmacophore models of ligands (Acharya et al., 2011); and 4) a structure-based approach, using the structure of the target molecule (Batool et al., 2019) (see Figure 1 for the drug development procedure). Among them, the structure-based approach, also known as the structure-based drug design (SBDD), offers several advantages, including rapid target identification/validation/lead identification, and efficient lead optimization, by focusing on drug-binding sites (Anderson, 2003; Kalyaanamoorthy and Chen, 2011). Moreover, by integrating computer-aided drug design techniques such as molecular docking-based virtual screening (Maia et al., 2020), molecular dynamics simulations (De Vivo et al., 2016), and machine learning (Bajorath, 2022), the SBDD workflow can be further accelerated (Sabe et al., 2021). Including computational tools, the specific processes and techniques requested in each step of the SBDD workflow are also described in Figure 1.

**FIGURE 2:**
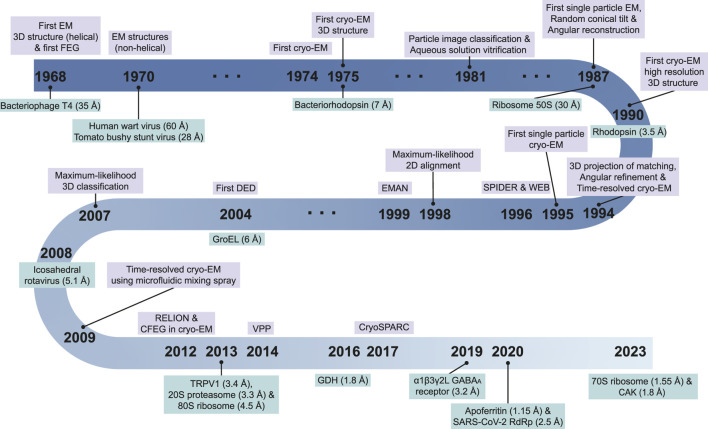
Electron microscopy (EM) timeline. The critical events in the development of techniques and software (top part) and determination of structures of critical biomacromolecules (bottom part) in EM research are marked by year. References and additional information on events are as follows: first EM 3D structure (sample: bacteriophage T4, 35 Å) (De Rosier and Klug, 1968) and first field emission gun (FEG) (Crewe et al., 1968) in 1968; first EM (sample: human wart virus, 60 Å; tomato bushy stunt virus, 28 Å) in 1970 (Crowther et al., 1970), first cryo-EM (diffraction image using the hydrated and frozen condition) (sample: catalase) in 1974 (Taylor and Glaeser, 1974); first cryo-EM 3D structure (sample: *Halobacterium halobium* rhodopsin, 7 Å) in 1975 (Henderson and Unwin, 1975); particle image classification in 1981 (van Heel and Frank, 1981); aqueous solution vitrification in 1981 (Dubochet and McDowall, 1981); random conical tilt and angular reconstitution (sample: 50S ribosomal subunit of *Escherichia coli*, 30 Å) in 1987 (Radermacher et al., 1987a); bacterial rhodopsin (cryo-EM structure at the highest resolution from diffraction data, 3.5 Å) in 1990 (Henderson et al., 1990); 3D projection of matching and angular refinement (sample: 70S *E. coli* ribosome, 37 Å) (Penczek et al., 1994); first time resolved cryo-EM (using on-grid mixing method) (sample: droplets) in 1994 (Berriman and Unwin, 1994); the first single particle cryo-EM (sample: 50S and 30S with tRNA, 25 Å) in 1995 (Frank et al., 1995); SPIDER and WEB in 1996 (Frank et al., 1996); maximum likelihood 2D alignment in 1998 (Sigworth, 1998); EMAN in 1999 (Ludtke et al., 1999); first direct electron detector (DED) as active pixel sensor chip in 2004 (Xuong et al., 2004); time-resolved cryo-EM as ms level (using microfluidic mixing-spray) (sample: *E. coli* ribosome 70S) in 2009 (Lu et al., 2009); RELION (the most popular software) (Scheres, 2012) and cold field emission gun (CFEG) for cryo-EM (Ricolleau et al., 2012) in 2012; TRPV1 (first membrane channel, 3.4 Å) in 2013 (Cao et al., 2013; Liao et al., 2013); 20S proteosome (*Thermoplasma acidophilum*, 3.3 Å) in 2013 (Bai et al., 2013); 80S ribosome (*Saccharomyces cerevisiae,* 4.5 Å) in 2013 (Li et al., 2013); Volta phase plate (VPP) in 2014 (Danev et al., 2014); glutamate dehydrogenase (GDH; the first achievement at a resolution below 2 Å, 1.8 Å) in 2016 (Merk et al., 2016); cryoSPARC (software using stochastic gradient descent and branch-and-bound maximum likelihood optimization algorithms) in 2017 (Punjani et al., 2017); α1β3γ2L γ-aminobutyric acid [GABA]_A_ receptor (target–scaffold complex structure using cryo-EM, 3.2 Å) in 2019 (Laverty et al., 2019); apoferritin (cryo-EM structure at the highest resolution, 1.15 Å) in 2020 (Yip et al., 2020); cyclin-dependent kinase (CDK)-activating kinase (CAK: a target for fragment-based drug discovery using cryo-EM) in 2023 (Cushing et al., 2023); 70S ribosome (*E. coli*, 1.55 Å) in 2023 (Fromm et al., 2023). The events for technical advancement are highlighted using purple and the cryo-EM structures are highlighted green.

**FIGURE 3:**
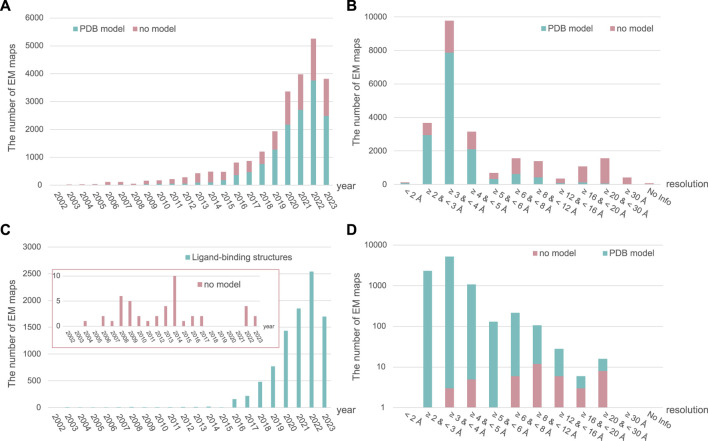
The annually released number and resolution distribution of EM maps in EM Data Bank (EMDB). The number of all released single-particle EM maps in the EMDB and structural models in the Protein Data Bank (PDB) from total samples are shown per year **(A)** and resolution range **(B)**. Ligand–target complex samples are also described per year **(C)** and resolution range **(D)**. The number of EM maps in EMDB with and without structural models in PDB is depicted in green and pink, respectively. All data for 2023 have been collected up to 2 August 2023.

In SBDD, obtaining high-resolution protein structures is crucial for identifying new ligand-binding sites and understanding molecular interactions between ligands and proteins (Lee et al., 2023). The traditional methods for obtaining high-resolution structural models include crystallography and nuclear magnetic resonance (NMR) spectroscopy. However, advancements in electron microscopy (EM) around the 2013 revolutionized research on structural biology, leading to the emergence of cryogenic EM (cryo-EM) (Fernandez-Leiro and Scheres, 2016; Herzik, 2020) (Figure 2: EM timeline). Cryo-EM has rapidly gained popularity and become a powerful tool for studying structures at near-atomic resolution (Callaway, 2020). As of 2 August 2023, almost 24,000 single-particle EM maps and 15,000 structural models have been deposited in the Electron Microscopy Data Bank (EMDB) and Protein Data Bank (PDB), respectively (Figures 3A,B). Furthermore, cryo-EM was successfully used to solve the structures of 52 antibody– and 9212 ligand–target complexes, including those of the small sized proteins (Figures 3C,D). It is critical to drug design for ligand-induced conformational changing targets. As shown in Figures 3A,C, the released number of EM maps has been increased annually with their model structures, and the number of ligand-binding complexes also has been increased in every year. The resolution of total EM maps was mainly distributed in the range of 2–5 Å (approx. 90% EM map coverage) (Figure 3B) and of approximately 80% of the complex EM maps were below 4 Å, a sufficient resolution for SBDD (Figure 3D). Currently, the highest reported resolution obtained using cryo-EM is 1.15 Å with human apoferritin (Figure 2) (Yip et al., 2020).

The critical advantages of cryo-EM over NMR or X-ray crystallography lie in several key aspects, such as, 1) cryo-EM allows the study of samples under near-physiological conditions, preserving the native state of the biomolecules; 2) Single-particle cryo-EM data provide structural heterogeneity of the target molecule, inferring its possible motions in native-like conditions; and 3) cryo-EM is applicable to a wide range of drug targets with different modes of action, making it a versatile tool for drug development. The features of cryo-EM are compared with those of X-ray crystallography, the dominantly applied approaches to determine structures for drug development in the aspects of specific advantages and disadvantages in Table 1. These indicated that cryo-EM has the potential to provide different views not covered by crystallography in drug development. Various technical advancements, including functionalized grids to resolve preferred orientation problem (Wang et al., 2020a; Fan and Sun, 2022; Lu et al., 2022), more powerful microscopes including sensitive detectors (Peplow, 2020; Frechin et al., 2023), and image processing software to remove noise (Sanchez-Garcia et al., 2021; Vilas et al., 2022), have enabled drug development using high-resolution cryo-EM. Furthermore, new techniques for the sample preparation of drug–protein complexes or drug screening using cryo-EM have been developed (Zhu et al., 2023). The advancements have paved the way for the utilization of cryo-EM in the drug discovery process and hold great promise for accelerating the development of new therapeutics.

**TABLE 1:** Comparison of techniques for structure determination.

		X-ray crystallography	Cryo-electron microscopy
**Sample**	**Sample size**	No size limit	Sample size limit (>100 kDa)^a^
	**Sample homogeneity**	Homogeneous samples with high purity	Heterogeneous samples possible
	**Sample amount**	0.2–2.0 μL of 5–50 mg/mL sample/well (total 1–100 μg)^b^	3 μL of 0.5–2 mg/mL sample/grid (total 5–15 µg)^c^
**Sample Preparation**	**Sample type**	Crystalline	Vitrified sample on the grids
	**Method to obtain sample**	Mixing samples with the optimal solution and incubation	Drop and vitrification of sample on the grids
	**Time required to obtain sample**	1 day–1 month for the crystal growth	Immediately after sample vitrification
	**Screening method to obtain sample**	High-throughput screening of crystal growth condition (e.g., solution component, temperature, pH and incubation time)	Grid screening for optimal distribution of single particles, various orientations, and optimal ice thickness
	**Screening scale**	1,000 < conditions of solution	<10 conditions of grid
	**Time required for screening**	2 min/96-well plate	1 h/grid
	**Time-resolved analysis**	Using X-ray free electron lasers (XFEL) (∼20 fs <)	Using microfluidic mixing injector (∼5 ms <)
**Data Collection**	**Beam type**	X-ray	Electron beam
	**Data type**	Diffraction data from the crystal	Magnified image of specimen
	**Radiation damage**	Crystal distortion, thermal vibration, generation of radicals, and covalent bond-breakage of sample	Beam-induced sample motion^d^, generation of radicals, and covalent bond-breakage of sample
	**Time periods for data collection**	10–60 min/sample at a synchrotron	1 h–1 day/sample
**Data Processing**	**Duration**	5–30 min^e^	Time-consuming^f^
	**Data processing steps**	Data indexing and scaling	Particle-picking, 2D classification, and 3D classification^g^
	**Resolution** ^h^	Highest distribution of 1.5–2.0 Å in PDB	Highest distribution of 3.0–3.5 Å in PDB and 3.0–4.0 Å in EMDB
	**Technique limitations**	Model-building limitation from flexible conformation	Model-building limitation from flexible conformation
	**Data file size**	<3 GB	>1 TB

^a^ The size limitation is occurred by the low signal-to-noise ratio. Scaffolds (e.g., fabs, megabodies, and symmetric proteins) and Volta phase plates (VPPs), have been used to overcome this limitation.

^b^ This amount is corresponded to one drop in each well.

^c^ This amount is estimated as the preparation of a 100 kDa macromolecule on a grid.

^d^ This can be reduced by the motion-correction algorithm and grid screening.

^e^ Data processing was performed using automated software.

^f^ Deep learning-based software was developed.

^g^ Multiple conformations were obtained during data processing.

^h^ Statistical parameters were obtained from the data since 2020.

Accelerating the practical and frequent application of cryo-EM for SBDD requires a comprehensive review of technical improvements in this field and successful case studies using cryo-EM for drug development. Recently, several papers have reviewed cryo-EM-based drug development from various perspectives (Subramaniam et al., 2016; Venien-Bryan et al., 2017; Garcia-Nafria and Tate, 2020; Van Drie and Tong, 2020; Wigge et al., 2020; de Oliveira et al., 2021; Lees et al., 2021; Aplin et al., 2022; Robertson et al., 2022a; Zhu et al., 2023). One review introduced the potency of cryo-EM applicable to drug development using protein structures with inhibitors (Subramaniam et al., 2016). Similarly, Renaud et al., 2018 summarized the crucial milestones in the cryo-EM timeline, including advancements in structure and technical development. A review by Skiniotis and colleagues mentioned new types of structures using cryo-EM, the advantages of cryo-EM, and technical improvements (Robertson et al., 2022a). Their comprehensive review highlighted the progress made in cryo-EM and its applications in SBDD. Furthermore, the workflow in cryo-EM studies, ranging from sample preparation to model building, was discussed. Research cases that focused on the structures of small molecules identified in protein–drug complexes using cryo-EM have also been reviewed (Subramaniam et al., 2016). However, despite the growing number of applications of cryo-EM in drug discovery (Figure 3), most previous reviews have mainly focused on considerable improvements in EM resolution in SBDD, whereas discussions on the technical achievements in drug discovery using cryo-EM are lacking.

In addition, considering the rapid growth of experimental research improvements in high-resolution cryo-EM and the accumulated examples of cryo-EM-based drug development, updated information on cryo-EM data-based drug design and practical aspects that aid in the acquisition of high-resolution images is required. In this review, we describe the general concepts and procedures of SBDD (Section 2), advanced techniques for cryo-EM-based structure identification (Section 3), and recently developed techniques for drug discovery (Section 4). In Section 5, the successful cases of cryo-EM-based drug design are presented. Finally, future perspectives and conclusions are discussed. This review contributes to enhancing the utility of cryo-EM in drug discovery and may lead to breakthroughs in the development of therapeutics.

## 2 Structure-based drug design

The origin of SBDD dates back to the 1970s (Brown and Shotton, 2015). The first target of SBDD was hemoglobin (Beddell et al., 1976). Based on the known structure of hemoglobin, artificial hemoglobin ligands were designed to bind and stabilize deoxyhemoglobin, thereby promoting oxygen release similar to that of its natural ligand, 2,3-diphosphoglycerate. Other well-known FDA-approved drugs that were developed using SBDD include dorzolamide (Biollaz et al., 1995), imatinib (Kantarjian et al., 2002; Szczepanek et al., 2022), and vemurafenib (Chapman et al., 2011; Flaherty et al., 2011). Subsequently, the first molecular docking algorithm was developed to understand ligand–target interactions (Kuntz et al., 1982). SBDD refers to the utilization of structural information of macromolecules acquired using either experimental or computational modeling methods (Ferreira et al., 2015). Understanding how ligands bind, activate, or inhibit their targets has great potential in the pharmaceutical industry, enabling the design of more efficient therapeutics (Whitesides and Krishnamurthy, 2005).

The typical drug development process comprises five main stages: 1) discovery and development, 2) preclinical research, 3) clinical trial, 4) FDA review and 5) FDA post-market safety monitoring (The United States Food and Drug Administration, 2018). With focus on the discovery and development phase, discovery and development can be further divided into five steps after target identification: 1) determination of the target structure, 2) identification of the target-binding sites/pockets, 3) identification of the hit compounds by *de novo* design or docking of existing virtual libraries, 4) *in vitro* validation, and 5) hit/lead optimization (Anderson, 2003; Shaker et al., 2021; Rakshit et al., 2022) (Figure 1). All these steps are described in the SBDD section of Figure 1. The first step in the SBDD workflow involves the determination of high-resolution structures of targets and/or target-ligand complexes after potential target identification and is considered the most important step (Batool et al., 2019). Protein structure can be determined using experimental or predicted using computational techniques. Experimental techniques include X-ray crystallography and NMR, which were widely used until the “resolution revolution” of cryo-EM, which has since gained popularity among structural biologists (Kuhlbrandt, 2014a; Kuhlbrandt, 2014b; Shoemaker and Ando, 2018). Especially, structures of membrane proteins, almost 30% of the human genome, are difficult to be determined using X-ray crystallography, as only approximately 2% of the deposited crystal structures in PDB are membrane proteins (Overington et al., 2006; Arinaminpathy et al., 2009; Kozma et al., 2013; Yin and Flynn, 2016; Jelokhani-Niaraki, 2022). However, with advancements in cryo-EM, the number of membrane protein structures solved using cryo-EM has increased annually, surpassing the number of crystal structures of membrane proteins in 2019 (Choy et al., 2021). If obtaining the target protein structure using experimental methods is challenging or unsuitable, computationally predicted models such as homology modelling and protein threading are applicable (Sliwoski et al., 2014; Leelananda and Lindert, 2016). Artificial intelligence-based software has also been used for model prediction, such as AlphaFold (Jumper et al., 2021; Bryant et al., 2022) by DeepMind, RoseTTAFold (Baek et al., 2021) by Baker’s group, and ESMFold (Lin et al., 2023) by Meta-AI. After obtaining the structural information, the second step involves the identification of binding pockets, which are areas that allow ligand binding, leading to the desired effect or action. Target–ligand interaction is identified by analyzing the interaction energies, electrostatic forces and van der Waals forces using algorithms or experimental data (Du et al., 2016). The third step is hit discovery, which is mainly performed using two methods: *de novo* design and virtual screening (Chávez-Hernández et al., 2023; Stanley and Segler, 2023). In the virtual screening approach, millions of drug-like compounds are docked to a target using computer algorithms and ranked according to their binding affinity (Lionta et al., 2014). Highly ranked compounds are then tested *in vitro*. In contrast, *de novo* drug design involves rationally designing chemical compounds with high affinity and target specificity using the receptor/target structure (Mouchlis et al., 2021). In the next step (step 4), the selected hits with the best scores are experimentally analyzed for functional activities *in vitro* to determine the most potent molecule. Their potency as drugs must be validated by measuring their target–hit affinities. The functional regulation of targets by hit molecules is also evaluated using cell-based assays. Finally, the hit molecules are optimized based on biophysical, biochemical, and cellular verification. In this process, the initial hits are optimized and developed into leads. In particular, repeated cycles of four steps are required for optimization: 1) identification of target–lead complex structures, 2) structure-based 3D-QSAR and pharmacophore analyses, 3) chemical synthesis of the newly designed molecules, and 4) activity assays.

In the next section, we describe the key technical achievements in high-resolution structural determination of drug targets using cryo-EM (Figure 2).

## 3 Technical advancements in cryo-EM for high-resolution structural determination

The initial EM was used to collect micrographs using electron beam. As shown in Figure 2, using micrographs obtained from the negatively stained T4 phage, its helical model was reconstructed in 1968 (De Rosier and Klug, 1968). Beyond the helical structure, which requires a single view for three dimensional model construction (De Rosier and Klug, 1968), in 1970, the three-dimensional structures of icosahedral (non-helical) viruses, negatively stained, were reconstructed using combining of micrographs acquired by using EM (Crowther et al., 1970). After these achievements, it was necessary to determine the structure of the biological samples without chemical modifications, such as fixation and staining. To overcome radiation damage caused by electrons and keep the biological sample intact, in 1974, the cryo-EM method was proposed (Taylor and Glaeser, 1974), and the following year, the structure of the rhodopsin protein was determined at 7 Å resolution from 18 micrographs and 15 diffraction images using cryo-EM (Henderson and Unwin, 1975). After 2 years, single particle EM analysis was developed, and the structure was identified from negative-stained *E. coli* 50S subunit at 30 Å resolution (Radermacher et al., 1987b). Finally, in 1990, Henderson et al., 1990 successfully determined the structure of bacteriorhodopsin using 72 micrographs of the near-atomic level of resolution (3.5 Å). In 1995, the potential of the single-particle analysis (SPA) method of cryo-EM to obtain asymmetric structural models using *Escherichia coli* 70S ribosome sample was validated (Frank et al., 1995).

After the first success in structure determination using the SPA technique, the continuous development of cryo-EM allowed the following applications: 1) SPA, pertaining to purified samples, 2) cryo-electron tomography (cryo-ET), which allows sample visualization in native environments (*in situ*), and 3) electron crystallography, such as microcrystal electron diffraction (microED) and 2D electron crystallography (Nannenga and Gonen, 2019). Especially, various revolutionary technical innovations have been developed for high-resolution structural determinations in SPA. In-ine with the overall workflow for structure determination using cryo-EM, this section consists of four subsections: 1) sample preparation, 2) grid optimization, 3) data collection, and 4) data processing, model building, and refinement. In each section, key representative technical improvements are explained, and structures determined using cryo-EM that are deemed important in historical, scientific, or resolution-related contexts are introduced in Figure 2 (cryo-EM structures).

### 3.1 Sample preparation

Sample preparation is the first and most important step in cryo-EM because the purity and quality of the sample directly affect the cryo-EM map quality (Passmore and Russo, 2016). In Section 3.1, three applicable techniques are described in the sample preparation step: 1) sample condition screening, 2) techniques for membrane proteins, and 3) techniques for small (<50 kDa) proteins.

Owing to limited instrument accessibility and time-consuming nature of ensuring good data quality in cryo-EM, it is necessary to evaluate the sample quality before collecting and analyzing cryo-EM data. Various factors influence the quality of single particles, such as pH, salt concentration, storage conditions, sample concentration, and purification steps (e.g., size exclusion chromatography) (Kampjut et al., 2021). For this reason, classical methods such as gel electrophoresis, size exclusion chromatography and dynamic light scattering (DLS) are required to scan and evaluate sample quality at the points of purity, sample heterogeneity of oligomeric states, sample aggregation and stability of complex state. Additionally, the analysis of sample quality using negative staining transmission electron microscopy (TEM) is a powerful and fast tool to estimate the adsorbed conditions of samples on the grids visually for parameters such as particle distribution (Scarff et al., 2018; Gonen, 2021).

Membrane proteins comprise a protein class that has benefited most from improvements in cryo-EM. To stabilize the membrane proteins, detergents and membrane mimetics are required. Autzen et al., 2019 mentioned various approaches in their review. One approach, introduced in the review, involves using detergents such as *n*-dodecyl-*β*-*D*-maltopyranoside, maltose neopentyl glycols, digitonin and glycol-diosgenin. Cholesterol derivatives, including cholesteryl hemisuccinate and CHAPSO, are also widely used in studies on membrane proteins. Another membrane-mimetic approach involves the use of amphipols, short hydrophilic protein polymers with hydrophobic side chains that cover the hydrophobic sites of membrane proteins (Tribet et al., 1996). The membrane proteins with amphipols are free from the thick belt near the transmembrane domain formed by detergents and nanodisc, one of the obstacles to determine membrane protein structure (Venien-Bryan and Fernandes, 2023). In that point, using amphipol is beneficial in the refinement process, the last stage of structure determination using cryo-EM. In addition to the detergents and amphipols, membrane scaffold proteins have been used to stabilize lipid bilayers by forming various lipid nanodiscs with membrane proteins (Denisov and Sligar, 2016; Efremov et al., 2017). Nanodiscs are prepared as follows: 1) incubation of detergent-solubilized proteins, lipids, and membrane scaffold proteins and 2) removal of detergents. In the procedure, selection of appropriate membrane scaffold proteins is critical to mimic the physiological status of membrane proteins. In 2019, Ognjenovic *et al.* published a review in which almost 50 structures of membrane proteins including ion channels, transporters, receptors, and others were determined using detergents, amphipols, and nanodiscs at a resolution above 4 Å (Ognjenovic et al., 2019). One of the recent cases with high resolution is the structure of the SARS-CoV-2 3a, a non-selective cation channel and a regulator of viral pathogenesis, determined at 2.1 Å resolution using lipid nanodiscs (Kern et al., 2021).

Although many advancements have been made in the field of structural biology in recent years, small protein structures remain challenging targets because of their inadequately distinguishable structural characteristics and low signal-to-noise ratios (Yeates et al., 2020; Zhang et al., 2022). By 2 August 2023, 9,872 EM maps released in the EMDB were from targets over 100 kDa, but only 117 (≈1.1% of the total) were from targets below 50 kDa. The 117 EM maps included models that were obtained by focused refinement of large molecules; the number of targets below 50 kDa was lower. To overcome this challenge, the strategy of increasing the molecular weight of target proteins is widely applied using scaffolds composed of an adaptor-specific scaffold core (or platform base) and a target-specific adaptor (Yeates et al., 2020). Two approaches have been reported for the development of adaptors: 1) designing a selective target-binding adaptor and 2) designing a fusion protein of an adaptor and target protein (Wentinck et al., 2022). Target-binding adaptors include antibodies and small antibody fragment-based proteins, such as antigen-binding fragments (Fabs) and nanobodies. The development of a fusion protein containing an epitope that the known antibody/nanobody/Fab recognizes with high specificity is another approach. By introducing the sequence from apocytochrome b_562_RIL (BRIL, ≈11 kDa) (Mukherjee et al., 2020; Tsutsumi et al., 2020), the glycogen synthase domain of *Pyrococcus abyssi* (PGS, ≈20 kDa) (Zhang et al., 2022) and the third intracellular loop from the kappa opioid receptor (κOR-ICL3) (Robertson et al., 2022b) to GPCRs, the target structures were determined using their specific nanobodies and Fabs (Wentinck et al., 2022; Zhang et al., 2022). The inserted regions such as BRIL, PGS, and κOR-ICL3 were developed as crystal chaperones, and also have been used for structure determination by cryo-EM (Chun et al., 2012; Mukherjee et al., 2020; Zhang et al., 2022; Miyagi et al., 2023). For instance, the BRIL sequence was inserted into the N-terminal region of solute carrier family 19 member 1 (SLC19A1) (65 kDa) with its substrate 5-methyltetrahydrofolate (5-MTHF) (Dang et al., 2022). The structure of the BRIL-containing SLC19A1–5-MTHF complex was determined at 3.5 Å resolution. In addition to adaptor proteins, scaffold cores, such as glutamine synthetase (dodecamer, D6), have been applied together with adaptors to increase the size by oligomerization of adaptor–target complexes (Coscia et al., 2016).

### 3.2 Grid optimization

After obtaining high-quality purified specimens, grid optimization is required to obtain vitrified samples on an EM grid with an appropriate ice layer thickness, the most time-consuming process (Benjin and Ling, 2020; Xu and Dang, 2022). Generally, on the gold or copper grid containing open holes, 3 µL of a purified sample is placed to form a thin layer ideally <100 nm thickness in the holes (Chua et al., 2022). The excess sample on the grid surface is then gently blotted using filter paper, and the grid is rapidly frozen in liquid propane or ethane cooled with liquid nitrogen for vitrification (Liu and Wang, 2023). Following this procedure, the optimal single-particle grid for cryo-EM is prepared as a single-particle state in the holes but away from the air–water interface (AWI). The adsorption of single particles on the AWI results in an orientation bias and/or partial or full denaturation of the drug targets by exposure of less hydrophilic sites to the AWI (Klebl et al., 2020).

In the grid preparation, various factors affect a single-particle grid, such as the grid material, glow discharge process, incubation conditions of proteins, and blotting protocols (Weissenberger et al., 2021). After blotting, the residual liquid layer on the grid bar or film is recruited, which affects the sample behavior (Glaeser, 2018). In some cases, these factors lead to disassembly, denaturation, and aggregation of macromolecules, particularly proteins, during sample preparation on the grid (Drulyte et al., 2018).

The ice thickness and AWI of single-particle cryo-EM grids are crucial in two respects: the orientation and the overlap of particles (Noble et al., 2018). Ice, which is thicker than the major axial length of a single particle or the minor axial length with at least an additional space of 20 nm, is required for the random orientation of particles. Otherwise, the particles are aligned near the AWI with a biased orientation distribution. In contrast, sufficiently thin ice is required to eliminate the overlap of multiple particles in the beam direction. Several methods have been developed to prepare ideal single-particle cryo-EM grids, such as the use of additives to form a protective layer during AWI (Chen et al., 2019), on-grid supports (Han et al., 2020), and vitrification devices to shorten the freezing time (Ravelli et al., 2020).

First, amphipathic molecules are the most common additives that naturally block AWI and enable the formation of randomly oriented particles. For example, CHAPSO, a zwitterionic detergent, solves problems related to the destabilization, aggregation, and/or preferential orientation of most specimens (Chen et al., 2022). For example, Ye et al., 2022 solved the structure of SARS-CoV-2 omicron spike protein ectodomain at 3 Å resolution using, but not being limited to, CHAPSO. They showed that the trimeric receptor binding domains (RBDs) of omicron spike proteins were mainly in the open (“standing up”) conformation, one of three RBDs up and the others down, as ready state for receptor binding.

Second, to overcome the AWI issue, the use of grids with carbon supports is suggested as another strategy in the grid preparation step to enhance image quality. Generally, two major supports are used: amorphous carbon-based and graphene-based supports (Han et al., 2020; Glaeser, 2021; Patel et al., 2021). Coating method with continuous thin films on grids can provide separate interaction surfaces, rather than AWI in “open hole” grids, for the target molecules. Instead of holes, the particles are distributed on the supports coated on a copper or gold grid as the film-water interface. Amorphous carbon films are commonly used to coat grids of large macromolecules (>300 kDa) (Grassucci et al., 2007). The amorphous carbon films are typically the first choice for challenging samples and may cause notable levels of background noise during imaging (Russo and Passmore, 2014). Graphene is a thin 2D nanomaterial that has been demonstrated to be an effective solution to reduce background noise, uneven particle distribution, and ice thickness (Naydenova et al., 2019a; Han et al., 2020).

The development of the vitrification process began in the 1980s with water (Dubochet and McDowall, 1981) (Figure 2). Using vitrification devices, rapid freezing (on the scale of millisecond or less) is expected to minimize the interaction of molecules with AWI by reducing the diffusion of particles to inhibit AWI (Klebl et al., 2020). Vitrobot (Thermo Fisher Scientific) and GP2 (Leica Microsystems) are commonly used plunge freezers. VitroJet, developed by Ravelli et al., 2020, enables the vitrification time to decrease to as low as 80 ms by automating grid preparation by placing samples on grids for freezing. VitroJet requires sub-nanoliter amounts of sample to be applied onto a grid using the pin-printing method, which is then immediately cooled with a cryogen, eliminating the blotting step. Similar to the VitroJet, the Spotiton is another grid-preparation device that has been shown to decrease sample vitrification time to approximately 10 ms by combining voltage-assisted spraying and vitrification (Jain et al., 2012; Razinkov et al., 2016; Kontziampasis et al., 2019).

### 3.3 Data collection

The next stage in the cryo-EM workflow is data collection using a cryogenic TEM (cryo-TEM). Electron microscopes are composed of five main parts: 1) an electron-beam-producing source, 2) a group of magnetic lenses, 3) a vacuum system, 4) a cryogenic sample holder, and 5) a detector for image capture (Chua et al., 2022). Since their introduction into the cryo-EM field, these microscope components have undergone major changes and have been developed to enable the collection of high-resolution structural data (Figure 2).

With the development of EMs, electron energy sources have improved to enhance the spatial and temporal coherence of electron beams from the initial energy sources such as tungsten filaments and LaB_6_ crystals (Joy, 2019). As LaB_6_ cathodes have narrower tips than tungsten filaments, they generate smaller radius electron beams with better coherence (Tang et al., 2021). Subsequently, the advanced electron guns, field-emission guns (FEGs) were developed in scanning electron microscope (Figure 2) (Crewe et al., 1968; Tonomura, 2011). Then, TEMs equipped with cold FEGs (CFEGs) were developed (Ricolleau et al., 2012). The CFEGs produced electron beams with an energy spread of approximately 0.3 eV, maintaining beam brightness when operating near room temperature (Hamaguchi et al., 2019). This energy spread is almost half that of the general FEG, resulting in a better signal-to-noise ratio owing to the improved coherence of the electron beam (Ricolleau et al., 2012; Kato et al., 2019).

With the development of the CFEGs, an ultrahigh vacuum (10^−8^–10^–9^ Pa) system is required for cryo-EM (El-Gomati et al., 2021). The ultrahigh vacuum system removes vaporized water from the vitrified sample and contaminants near the tip and prevents interference from the electron beam and induces electron diffraction by the contaminants and water molecules (Kogure, 2013; Cheng, 2015).

A phase plate introduces a phase shift in the diffraction plane of a microscope, resulting in phase contrast (Danev and Baumeister, 2016). Therefore, it improves the signal-to-noise ratio by providing enhanced image contrast and in-focus data acquisition (Glaeser, 2013). The phase plates have been used in light microscopes for a long time but were unavailable for use in electron microscopes until recently (Danev and Baumeister, 2016). The Zernike phase plate, a thin material film with a central hole and a phase shift of ∼π/2, has been used in cryo-EMs, although it has the disadvantages of a short lifespan and fringe artifact creation (Danev et al., 2009; Glaeser, 2013). Moreover, the difficulty in aligning the beam center to a small hole is a barrier to the use of Zernike phase plate. Therefore, a new phase plate, the Volta phase plate (VPP), was introduced in the field of EM for biological samples in 2014 (Danev et al., 2014). The VPP is a continuous amorphous carbon film with working conditions of approximately 25 mA and 200°C to prevent contamination. It has no fringe artifacts, a long lifespan, and no alignment requirements. Using the VPP, the full-length calcitonin receptor (CTR), a class B GPCR was reconstructed in a complex with peptide agonist and Gαβγ heterotrimeric protein, as a therapeutic target for several bone diseases (Liang et al., 2017). In addition to thin film-based phase plates such as Zernike phase plate and VPP, two new types of phase plates, magnetic phase plates using a magnetic field and laser phase plates using photon–electron interactions, have been developed (Wang and Fan, 2019). Very recently, the laser phase plates was applied to cryo-EM; however, it has not yet been commercialized (Axelrod et al., 2023).

Aberration correctors are another critical component for improving the power of cryo-EM for high-resolution structures. (Evans et al., 2008). There are two major aberrations: spherical aberration caused by failure of convergence of the paraxial ray and marginal ray passing through the objective lens, and chromatic aberration caused by various wavelengths of the electron beam. The spherical aberration was removed using correctors and aspherical lenses (Fan et al., 2017). To remove chromatic aberration with a FEG and a monochromator, a chromatic aberration correctors composed of an electromagnetic hexapole or quadra/octa-pole was also applied (Leary and Brydson, 2011). Eliminating these aberrations using correctors enhances the image quality of the focused beam (Freitag et al., 2005).

Photographic films, charge-coupled device (CCD) detectors, and complementary metal oxide semiconductors (CMOSs) were routinely used for image detection and recording in EMs. Photographic films have the advantage of collecting images of a large size, but require additional processes, such as digitizing the acquired images for further processing and analysis. Compared with films, CCD and CMOS detectors offer automated data acquisition and immediate image access for analysis. Usually, to collect images in these detectors, a single instant electron is scattered by scintillators, releasing photons. The photons passing through the optical fibers are then detected by a CCD or CMOS. In this procedure, photons generated from scattered single instant electrons reduce the resolution of spatial information and decrease signal-to-noise levels by noise generation (Meyer and Kirkland, 1998). Additionally, the image quality obtained from detectors coupled with scintillators and fiber optic plates is appropriate at 100 keV, whereas the noise increases at high powers, such as 200 and 300 keV, which are the general energies of cryo-EM (Bammes et al., 2012; McMullan et al., 2014). Although CMOS detectors have advantages over CCD detectors, such as faster frame rates and lower blurriness, problems caused by using scintillators and optic fibers remain. A new technique, the direct electron detector (DEDs), was designed in 2004 and applied to cryo-EM (Xuong et al., 2004; Bai et al., 2015). There are two types of DEDs: 1) monolithic active pixel sensors (MAPS) (Peric, 2007) and 2) hybrid pixel array detectors, including Medipix sensor-based detectors (Jakubek et al., 2004) and electron microscope pixel array detectors (Tate et al., 2016). Among these, MAPS exhibited the best performance at high electron beam energies. Therefore, MAPS are widely used in cryo-EM at 200 and 300 keV. In DEDs, unlike previous detectors, MAPS rather than scintillators and optic fibers, transfer electrons to detectors directly, and DEDs enable the detection of electrons with high resolution and high signal-to-noise ratio by reducing noise and reducing the loss of signal in the electron-counting mode (Levin, 2021). Moreover, DEDs exhibit a high frame rate when collecting images. This improvement in detectors leads to the resolution revolution in cryo-EM.

Using high-voltage (300 keV) cryo-TEMs routinely, protein structures were determined at high resolution. With other technical advancements, including FEGs and DEDs, the low-voltage microscopes such as Glacios (Thermo Fisher Scientific, MA, United States) and CRYO ARM 200 (JEOL Ltd., Japan) are also used for structure determination (Merk et al., 2020; Wu et al., 2020; Koh et al., 2022; Thangaratnarajah et al., 2022). Various studies have shown membrane protein structures at 2–4 Å resolution using 200 keV microscopes (Fan et al., 2020; Oosterheert and Gros, 2020; Cao et al., 2021; Budiardjo et al., 2022; Zarkadas et al., 2022). As an example, the inhibited and the active states of human mitochondrial calcium uniporter (MCU) holocomplex structure, comprised of four main proteins: MCU, mitochondrial calcium uptake 1 and 2, and an essential MCU regulator were determined at 3.6 Å resolution (Fan et al., 2020). The more advanced 100 keV cryo-EM was also developed and applied to determine several structures with diverse sizes in 2019 (Naydenova et al., 2019b). They reported structures of macromolecules in the range of 64 kDa–4.5 MDa size within 3.4–8.4 Å resolutions. Later, using the 100 keV cryo-EM, macromolecules with size range between 140 kDa and 2 MDa such as 70 S ribosome and GABA_A_ receptor were structurally determined within 2.7–4.5 Å resolution range (McMullan et al., 2023). The development of low energy cryo-EM enhanced the user accessibility to cryo-EM while increasing the number of available cryo-EM and reducing costs associated with the equipment and its installation and maintenance (A low-cost electron microscope maps proteins at speed, 2023). Thus, it is expected that the lowered accessibility barriers will accelerate determination of targets structures for SBDD.

### 3.4 Data processing, model building, and refinement

Cryo-EM is differentiated from X-ray crystallography by using “images” instead of diffraction patterns as primary data (Wang and Wang, 2017). A typical image-processing workflow for cryo-EM data can be divided into five steps: 1) data pre-processing, 2) particle picking and extraction, 3) 2D classification, 4) 3D reconstruction, and lastly 5) 3D refinement (Lyumkis, 2019; Chung et al., 2022). After releasing SPIDER and WEB in 1996 (Frank et al., 1996), several software packages are available for the processing steps, including EMAN (Ludtke et al., 1999; Tang et al., 2007; Bell et al., 2016), RELION (Scheres, 2012; Zivanov et al., 2018), cryoSPARC (Punjani et al., 2017), and cisTEM (Grant et al., 2018).

After collecting the images, data pre-processing is initiated by correcting the images of particles moved by electron beam exposure. Most particle movement occurs for a very short time in the early period of beam exposure, after which the movement decreases spatially and temporally (called stable but not fixed) (Li et al., 2013). Therefore, rejection of the images obtained during the early period was suggested as a solution to remove the blurriness of the images due to beam-induced particle motion. However, this strategy was not always welcome because the longer the exposure to the sample, the more radiation damage accumulated in the samples, as shown in Table 1. This dilemma has been solved through the development and advancement of DEDs, as mentioned in Section 3.3. The high frame rate (10–400 frames/s) of DEDs allows images to be captured as multi-frames, such as a movie, in a single exposure. Although it produces a low signal and low-resolution contrast by short exposure at the millisecond level, high-resolution images can be obtained. Moreover, a high frame rate enables the collection of images that are stable and undamaged by beam exposure (Li et al., 2013; Zivanov et al., 2019). Therefore, using images collected from the least damaged particles in the early frames is more advantageous despite the blurriness caused by the high movement of particles (Cheng et al., 2015). Software has been developed in addition to cameras. Software such as MotionCor2 (Zheng et al., 2017) and *alignframes_lmbfgs* (Rubinstein and Brubaker, 2015) were developed to estimate and correct frame motion using motion correction algorithms, resulting in motion-corrected images. To assess the quality of the micrographs, contrast transfer function (CTF) estimation is performed. CTF obtained using software such as CTFFIND4 (Rohou and Grigorieff, 2015) and Gctf (Zhang, 2016) is required to curate micrographs based on the estimated defocus and astigmatism levels.

The particle-picking step to locate the target molecules in micrographs is challenging for several reasons, such as low signal-to-noise ratios, impurities on the micrograph, non-uniform distributions, preferential orientations, and undistinguishable structural characteristics (Chua et al., 2022). Several groups suggested the required number of collected particles from micrographs to obtain high resolution EM maps, as “the more, the higher.” In 2016, Danev and Baumeister reported 5,000–10,000 particles were required to achieve below 3.5 Å resolution with Fourier shell correlation (FSC) = 0.5 criterion (Danev and Baumeister, 2016). However, Chua and the colleagues suggested that ≈1,000,000 particles were required to obtain structures at 3.5 Å or higher resolution with FSC = 0.143 criterion (Chua et al., 2022). They estimated the total number of requested micrographs as 5,000–10,000, which was acquired from division of ≈1,000,000 particles by the average number of analyzable particles per micrograph, ≈100 particles/micrograph.

The initial software for particle picking used user intervention, which was a time-consuming approach. The semi-automated pickers were developed based mainly on template-base method (applied into the particle picker in RELION-1.3 (Scheres, 2012; 2015)), feature-based method (applied to DoG Picker in Appion (using difference of Gaussian) (Voss et al., 2009), and Laplacian of Gaussian-applied picker in RELION (Kimanius et al., 2021) and Blob Picker in cryoSPARC (Punjani et al., 2017; Chung et al., 2022; Wu et al., 2022). The results obtained using semi-automated pickers were more efficient than those obtained using manual pickers, but the selected input images or features induced a bias. To avoid this bias, the convolutional neural networks-based particle pickers has been raised such as DeepPicker (Wang et al., 2016), DeepEM (Zhu et al., 2017), Topaz (Bepler et al., 2020), Warp (Tegunov and Cramer, 2019), crYOLO (Wagner et al., 2019; Wagner and Raunser, 2020), PIXer (Zhang et al., 2019), and DeepCryoPicker (Al-Azzawi et al., 2020). Their algorithms are composed of two steps: 1) a training network using manually selected sets of micrographs, and 2) picking particles automatically using trained algorithms.

In the third step, 2D classification is the process of discriminating and refining clearly aligned images of particles from other images, and grouping the particles based on conformation and composition for 3D classification. To cluster the similar particle images in the 2D classification step, the maximum-likelihood (Sigworth, 1998) or cross-correlation (CC) (Radermacher and Ruiz, 2019) approach is applied to align and average particles (Figure 2). Using the CC approach, images are aligned to acquire the maximum CC coefficient value between two images or between a single image and the average of the images. However, the CC approaches can generate high but false correlation coefficients, particularly in the images with low signal-to-noise ratios, which are usually obtained from the DED images. Unlike CC approaches, the maximum-likelihood approach calculates the probability-weighted averages of all possible orientations of images (Sigworth et al., 2010). With the hidden variables assumption, the maximum-likelihood algorithm has been broadly applied to software packages such as FREALIGN (Grigorieff, 2007), RELION (Scheres, 2012), and cryoSPARC (Punjani et al., 2017). The representative images acquired after 2D classification are again used as a training set for particle picking, and then picking particles and 2D classification followed as an iterative process to sort particles more thoroughly into a set of high-quality particles (McSweeney et al., 2020).

Three steps are required to obtain optimally estimated 3D EM maps from 2D particle images: 3D reconstruction, 3D classification, and refinement. Therefore, the three steps are integrated as described in the previous paragraph. Three-dimensional construction is performed using a known reference structure or *Ab initio* model (Joubert and Habeck, 2015). References can be obtained from databases (PDB and EMDB) or Random Conical Tilt-applied 3D models from negative-stained EM images. *Ab initio* models are constructed using various algorithms, such as the common-lines-based model, random-model methods, stochastic hill climbing, stochastic gradient descent, and Bayesian approach (Joubert and Habeck, 2015). For example, the stochastic hill climbing algorithm was used in SIMPLE/PRIME (Reboul et al., 2016; Reboul et al., 2018) and the stochastic gradient descent algorithm was implemented in the 3D reconstruction step in cryoSPARC (Punjani et al., 2017) and RELION (Scheres, 2012). One or more initial models are constructed from the reference structure by using a projection-matching algorithm (Nogales and Scheres, 2015). Using the initial models, an unbiased 3D classification is performed, consequently helping to identify multiple conformations or separate junk particles. Although the process of 3D classification is the same as that of 2D classification, it is critical to distinguish between the various conformations of the target molecules or the different complex structures with subtle differences (Murshudov, 2016).

Three-dimensional refinement is the final step in refining a 3D EM map to a high resolution, finding the optimal orientation for 2D particles using the initially reconstructed map in the previous step as a reference. The branch-and-bound algorithm (Lawler and Wood, 1966; Punjani et al., 2017; Zhong et al., 2021) and the adaptive expectation-maximization algorithm (Tagare et al., 2010; Scheres, 2012) have been applied to image alignment. Because refinement of the entire structure has the disadvantage of averaging 2D images from various conformations, several additional refinement methods for determining the structure of flexible parts have been proposed. Multibody refinement in RELION considers complexes as a group of independent rigid bodies and calculates the sum of the rigid bodies as a flexible complex (Nakane and Scheres, 2021). The 3D variability analysis (3DVA) implemented in cryoSPARC fits a high-resolution subspace model to a flexible map (Punjani and Fleet, 2021). CryoDRGN reconstructs 3D maps into the several classes using deep neural networks (Zhong et al., 2021; Kinman et al., 2023). Murshudov et al., 2011 reviewed software for model fitting (e.g., *Coot* (Emsley et al., 2010), Jiggle Fit (Casanal et al., 2020), and Morphing (Casanal et al., 2020)) and refinement (e.g., *ProSMART* (Nicholls et al., 2014) and *REFMAC* (Brown et al., 2015). To evaluate the model, a FSC curve is obtained by calculating the correlation between two subsets, each including an independent half of the complete image set (Murshudov, 2016). In the FSC curve, the resolution at FSC values of 0.143 and rarely 0.5 is defined as the resolution of the model (Rosenthal and Henderson, 2003). An additional sharpening step that specifies the B-factor value improves the EM map interpretability (Fernandez et al., 2008). The local resolution of the map is processed and visualized using UCSF ChimeraX (Goddard et al., 2018; Pettersen et al., 2021).

## 4 New techniques for cryo-EM–based drug design

**FIGURE 4:**
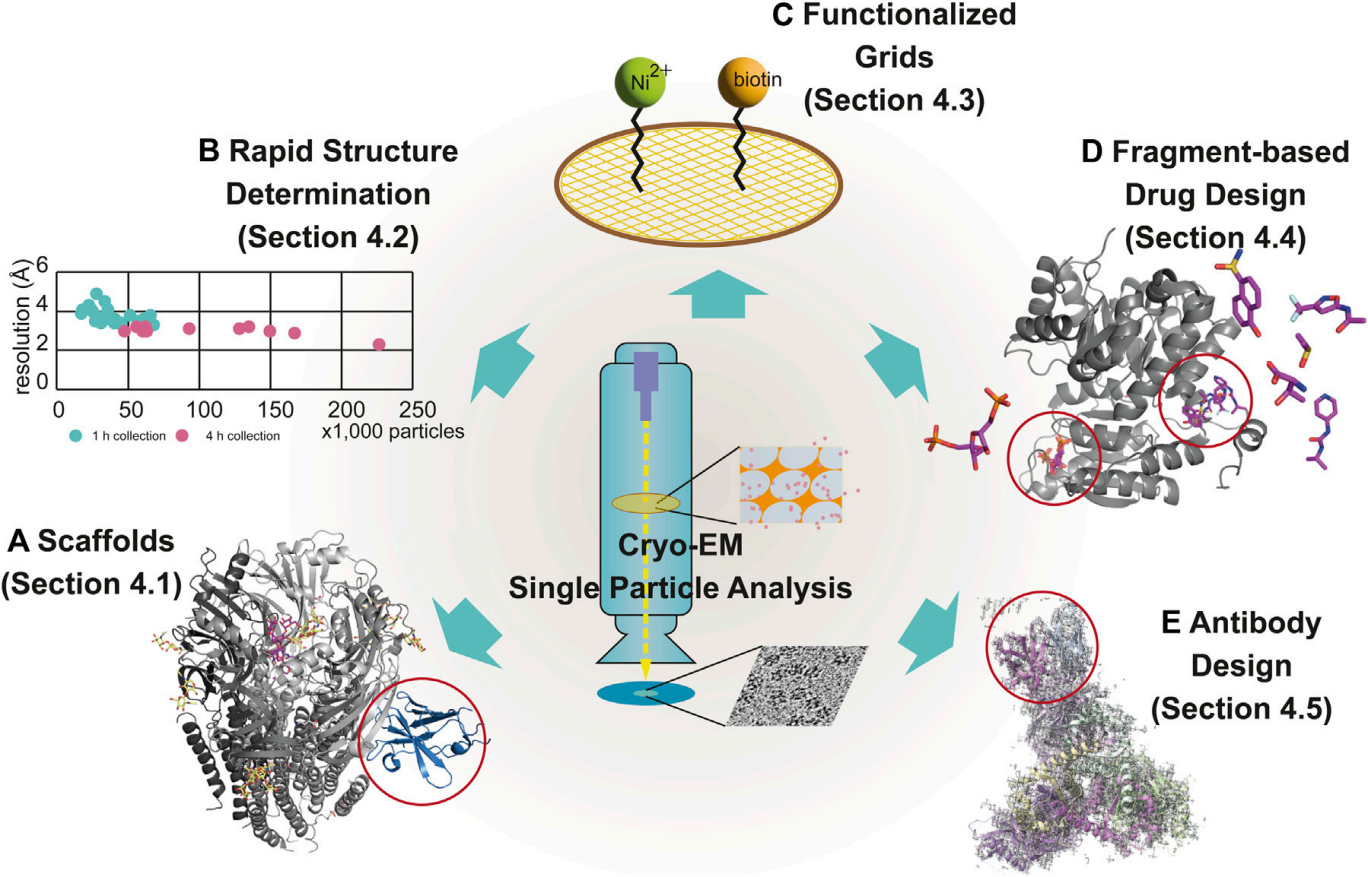
Modern techniques for cryo-EM–based drug discovery. The newly emerged techniques of cryo-EM for drug discovery are scaffolds **(A)**, rapid structure determination **(B)**, functionalized grids **(C)**, fragment-based drug design **(D)**, and antibody design **(E)**, described in Section 4.1, 4.2, 4.3, 4.4 and 4.5, respectively. Examples using each technique are displayed with individual EM maps and structural models. The following are representative examples: **(A)** α1β3γ2L GABA_A_ receptor (6HUJ:EMD-02779) (Fromm et al., 2023); **(D)** PKM2 (6TTI:EMD-10577, 6TTF:EMD-10575, 6TTH:EMD-10576, and 6TTQ:EMD-10584) (Saur et al., 2020); and **(E)** the trimer of the heterodimer of gp120 (BG505 SOSIP MD39) and gp41 (BG505 SOSIP MD3) with antigen-binding fragment (Fab) (Rh.33104 polyclonal antibody class C (7I8A:EMD-23227) (Antanasijevic et al., 2022a). Rapid structure determination is described using a scatter graph, comparing the resolution and the number of particles from 39 samples, collected for 1 h (n = 26) (green) and 4 h (n = 13) (pink) **(B)** (Cushing et al., 2023). The scaffold in **(A)**, fragments-binding sites in **(D)** and antibody in **(E)** are marked using red open circles.

The main bottleneck in the SBDD studies is the insufficient number of high-resolution structures of drug targets such as membrane proteins, which constitute 60% of all drug targets (Overington et al., 2006; Yin and Flynn, 2016). Most SBDD studies rely on X-ray crystallography, which makes it difficult to determine the structure of every molecule because of the difficulty of crystallization (Grey and Thompson, 2010). With the resolution revolution in cryo-EM, solving the high-resolution structures of difficult targets has become possible in the last decade. In this section, we discuss new techniques applied to SBDD using cryo-EM, including 1) scaffolds used for the determination of the cryo-EM structure of small-sized targets, 2) rapid determination of the structure of protein/ligand complexes, 3) functionalized grids for cryo-EM studies of drug targets at low concentrations, 4) fragment-based drug design (FBDD), and 5) antibody design (Figure 4).

### 4.1 Scaffolds used for determination of the cryo-EM structure of targets

As mentioned in Section 3.1, the use of scaffolds is an effective solution for increasing the size of biological samples and overcoming the problem of indistinguishable signals from noise in small targets. Early methods that used scaffolds in sample preparation for cryo-EM were the same as those used in crystallography: inserting a known epitope sequence into the target and forming an antibody/nanobody/Fab–target complex. However, improvements in antibody engineering techniques using computational approaches have allowed for the efficient design of target-specific antibody/nanobody/Fab and their application to the structural determination of target molecules. Recently, the use of the megabodies and Legobodies has been proposed as the most promising approach for cryo-EM studies. Additionally, ankyrin repeats are used in cryo-EM to prepare the target-specific scaffolds, called designed ankyrin repeat proteins (DARPins), by forming oligomers with scaffold cores. Scaffolds affect not only the target size but also the diversity of the orientation of the samples, and comprehensively enhance the resolution. In this section, we describe scaffolds and their applications in structural determination.

A BRIL-based scaffold was designed to reveal different states of GPCRs by various regulators using cryo-EM (Bernhard and Che, 2024). The mBRIL, the less flexible version of BRIL, was developed to reduce idiosyncratic behavior in BRIL-inserted GPCR (Guo et al., 2024). With the mBRIL, the extended cytosolic helix at C-terminus using ALFA tag (Gotzke et al., 2019) and the scaffolds between ALFA tag and mBRIL were applied to identify the complex structures of β2-adrenergic receptor with FDA-approved drugs, olodaterol and formoterol (Guo et al., 2024).

Laverty et al., 2019 developed the first megabody Mb38, the chimeric protein comprised two parts: 1) a specific nanobody to the α1-subunit of the γ-aminobutyric acid receptor subtype-A (GABA_A_ receptor) and 2) the scaffold protein from the extracellular adhesin domain of *Helicobacter pylor*i HopQ. By using Mb38, the structure of the full-length human α1β3γ2L GABA_A_ receptor was determined at 3.2 Å resolution in the lipid bilayer. Masiulis *et al.* also reported the five complex structures of the full-length human α1β3γ2L GABA_A_ receptor with picrotoxin, bicuculline, GABA, alprazolam, and diazepam in separate lipid nanodiscs using Mb38 (57 kDa) (Figure 4A) (Masiulis et al., 2019). In 2020, using the variant of Mb38, Mb25 (56 kDa), the structure of the human β3 homopentameric GABA_A_ receptor was identified at 1.7 Å resolution (Nakane et al., 2020). Later, the megabodies targeting homopentameric GABA receptors were further improved to increase the bulkiness of samples and reduce the biased distribution of orientation of membrane proteins (Uchanski et al., 2021). Other types of the megabodies have been developed to target small size of membrane proteins. The human sodium/bile acid cotransporter (NTCP, 36 kDa) was structurally identified using Mb91, a fusion protein of the nanobody with *E. coli* glucosidase YgjK (89 kDa) at 2.88 Å resolution (Goutam et al., 2022). Mb177, similar to Mb91, was also developed and used to determine the structure of human Hedgehog acyltransferase (HHAT, 58 kDa) at 2.7 Å resolution (Coupland et al., 2021).

Another approach for solving this problem is to use Legobodies. Legobody is an assembly of three components: 1) target-specific nanobody (≈14 kDa), 2) the nanobody-binding Fab (≈49 kDa), 3) the maltose-binding protein (MBP)-based fusion protein (MBP with nanobody-binding protein A domain C (PrAC); V_H_ (in Fab)-binding protein A domain D (PrAD); and C_H_ (in Fab)-binding protein G (PrG)), called MBP–PrAC–PrAD–PrG (59 kDa) (Wu and Rapoport, 2021). The MBP–PrAC–PrAD–PrG protein reduces the flexibility of target–nanobody–Fab trimer in solution and increases its size. By binding to the heterotrimeric Legobody, the target protein obtains approximately an additional 120 kDa of its original size. Wu and Rapoport, 2021 used this system to solve the structures of both endoplasmic reticulum (ER) lumen protein-retaining receptor 2 (KDELR2, ≈30 kDa) and SARS-CoV-2 spike protein receptor-binding domain (RBD, ≈25 kDa). The Legobody was also used to determine the structure of the inner mitochondrial membrane protein uncoupling protein 1 (UCP1). UCP1 is activated by fatty acids and small molecules such as 2,4-dinitrophenol, and negatively regulated by purine nucleotides, ATP, and GTP (Kang and Chen, 2023). Using Legobodies including newly screened UCP1-specific nanobody (called sybody 12F2), Kang and Chen identified the structures of UCP1 (32 kDa) as three different states: nucleotide-free, 2,4-dinitrophenol-bound, and ATP-bound. Although the model structures of the Legobodies are not shown in the models deposited in the PDB (8HBV, 8HBW, and 8J1N), their maps are clearly described in the EM maps in EMDB (EMD-34644, EMD-34645, and EMD-35928).

In addition to antibody-like proteins, engineered ankyrin repeats have been used to design target-specific binding partners (Li et al., 2006). Initially, based on the ankyrin repeat, a target-specific DARPin was screened from the DARPin library and used as a crystal chaperone and biosensor (Pluckthun, 2015; Boersma, 2018). Due to the small size of DARPin, it should be used with scaffold cores such as aldolase in structure determination using cryo-EM (Liu et al., 2019; Yao et al., 2019). The use of the DARPin–scaffold core fusion increases the sample size by binding of the DARPin–scaffold core to the target and by forming oligomers of targets, mediated by the scaffold core. The structure of green fluorescent protein (GFP, 26 kDa) was identified using aldolase-fusion DARPin (tetramer, D2) at 5–8 Å resolution (Yao et al., 2019). Later, improvement of the DARPin–nanocage fusion scaffold (called DARP14, heterotetracosamer as a dodecameric heterodimer of DARP14 subunit A (DARPin-conjugated form, 34 kDa) and subunit B (DARPin-free form, 14 kDa)) enhanced the resolution of GFP to 3.8 Å (Liu et al., 2018a; Liu et al., 2019).

### 4.2 Rapid determination of the structure of protein/ligand complex

SBDD studies require the screening of several designed drugs. Hence, cost-effective and rapid methodologies are important to render the process more efficient. As shown in Table 1, to overcome the disadvantages of the cryo-EM workflow, such as lower throughput of processes compared to crystallography, methodologies have been developed for more efficient processes, such as automation of sample preparation (Koning et al., 2022), data collection steps (Tan et al., 2016) and minimization of required image numbers for structure determination (Cushing et al., 2023).

One of the recent achievements is automation of data acquisition. Smart EPU Software is available for high-throughput data acquisition with autoloader-equipped cryo-TEMs (Drulyte et al., 2022). Using the autoloader, screening and collecting images from maximum 12 grids were available. It decreases the time consumed for sample loading and environment setting such as vacuum condition and temperature. The EPU software also contains a rapid data acquisition mode that allows unattended screening of multiple grids. The “Fast Acquisition” mode in EPU utilizes beam tilt instead of stage movement to each point. Neutralizing SARS-CoV-2 antibodies specific to the RBD domain were generated, and 12 Fabs with spike proteins in complex forms were selected and subjected to data collection for structure determination using the EPU software in 48 h. After data processing, twelve sub-3 Å structures of Fabs with spike protein were reconstructed. As well as EPU, SerialEM also uses beam-tilt compensation algorithm for fast image collection (Takaba et al., 2020). EPU, SerialEM and Leginon (Cheng et al., 2021) provide condition of holes and squares of sample grids, and allow to screen and collect data in promising area of grids, and finally reduce the time for data acquisition. *Ptolemy* software brought the full process of data collection by automation using machine learning and computer vision algorithms (Kim et al., 2023).

Cushing et al., 2023 tested the correlation between the number of images for 3D model reconstruction and resolution to determine the structures for SBDD. The cyclin-dependent kinase (CDK)-activating kinase (CAK) heterotrimeric protein complex was formed by CDK7, MAT1, and cyclin H and affected cell division and cell growth by regulating transcription initiation (Sava et al., 2020). Owing to its important role in cellular pathways, the CAK complex is a potential target for cancer drugs and antivirals (Hutterer et al., 2015). Cushing et al., 2023 recently reported the complex structures of CAK complex with 12 different inhibitors using the rapid sample screening strategy. They initially collected ≈500 particles/grid from 26 grids using protein complexes with 12 inhibitors for 1 h and selected 13 grids among them based on processed results including refinement. The selected 13 sets were subsequently used to collecting ≈2,000 particles/grid for 4 h. This strategy suggested that a small number of particles was enough to screen and select the samples for further process. Using the fast-screening strategy, they determined a dozen of structures at 3.5–4 Å resolution daily using only a 200 kV cryo-TEM (Figure 4B).

### 4.3 Functionalized grids for cryo-EM studies of drug targets at low concentration

In cryo-EM, samples are selected using the particle-picking process and used as input data. To exploit this advantage, the desired samples are selected and enriched using functionalized grids. For example, the grid was coated with affinity ligands and antibodies to capture samples (Llaguno et al., 2014; Yu et al., 2016). Functionalized grids successfully enrich the number of target proteins on the grid surface, enabling the use of low-concentration samples such as viral particles or membrane proteins, which are the main targets in SBDD studies, without the need to increase expression levels (Yu et al., 2016; Wang et al., 2020b).

For this purpose, high-affinity selective ligands, such as nickel–nitrilotriacetic acid (Ni–NTA) to His-tag, biotin to streptavidin, and antibodies to Protein A/G, have been successfully used as functional groups coated on the grid (Llaguno et al., 2014; Earl et al., 2017) (Figure 4C). Yu et al., 2016 used antibody-based affinity grids to solve the Tulane virus structure at 2.6 Å resolution using a low concentration of the virus. Using this approach, researchers detected the target sample bound to antibody–coated grids, suggesting that this approach is useful for target samples that are difficult to prepare and have low yields. Recently, Cheng et al., 2023 introduced dual-affinity graphene grids prepared with two ligands of different affinities: Ni-NTA and polyethylene glycol–biotin. By targeting different sites using dual-affinity graphene grids, they obtained 20S proteasomes. They also solved the spike protein of SARS-CoV-2. Dual-functionalized grids have higher specificity and affinity for target sample molecules than single-affinity-functionalized grids, leading to their balanced distribution on the grids. Therefore, the dual-affinity graphene grid has been claimed to be an effective approach for preparing ideal grids for structural determination of drug targets using cryo-EM.

More generally, functional groups were used to modify cryo-EM grids. Agard’s group applied amino/PEG-amino graphene oxide in their research (Wang et al., 2020b). Multifunctional graphene for cryo-EM grids, functionalized with amine, carboxyl, thiol and phenyl groups, were also developed (Naydenova et al., 2019b). Using the functionalized grids, Naydenova and colleagues successfully enhanced randomness of particles orientation, and determined structures of ribosome and apoferritin at high-resolution. In addition to non-covalent functionalization, Nickl et al., 2023 developed graphene-based grid employing covalent bonding to stabilize specimens.

### 4.4 Fragment-based drug design

SBDD studies rely on the identification of drug-binding pockets in proteins and ligands in the binding pockets (Figure 1). However, identification of ligand-binding pockets is difficult if the protein has undefined pockets (Shelke et al., 2010). To overcome this limitation, fragment-based approaches have been widely used in SBDD studies (Murray and Rees, 2009). Therefore, FBDD is effective for identifying novel ligand-binding pockets and developing potent drugs. In this approach, drug-like molecules are generated from small chemical fragments that bind to drug target proteins with low affinity (Li, 2020). Through structural studies of target proteins complexed with small compound libraries, target-bound fragments and their binding sites can be identified (Wang et al., 2023).

This method was initially developed for X-ray crystallography but was soon used in NMR-based drug screening. Cryo-EM has become a powerful tool for FBDD studies owing to technical advancements that allow the high-resolution of structures. In a recent study, Saur et al., 2020 showed that cryo-EM can be utilized for FBDD studies by identifying two different target proteins, β-galactosidase and pyruvate kinase M2 (PKM2) (Figure 4D). β-galactosidase is a homotetrameric protein with 465-kDa molecular weight that plays a role in the hydrolysis of lactose to glucose and galactose (Bartesaghi et al., 2015). The cryo-EM structure of β-galactosidase at a resolution between 2.2 and 2.3 Å was determined using three different fragment-sized ligands. The ligand structures at the binding site and the conformational changes were clearly detected in the density maps, indicating that cryo-EM has the potential to guide FBDD research. In another study, the capacity of cryo-EM for fragment screening was demonstrated using an oncology target, PKM2, which is involved in the conversion of phosphoenolpyruvate to pyruvate. PKM2 is a potential therapeutic target because of its involvement in cancer development (Zhu et al., 2021). To demonstrate the applicability of cryo-EM for fragment screening, 68 different ligands were used at high concentrations to form complexes with PKM2. The structures of these complexes were solved after 68 days of data collection, followed by 2–3 days of data acquisition depending on the sample. By preparing these structures, they indicated that a 3.2 Å resolution was adequate for determining the binding modes of fragment-sized molecules and clearly showed that the fragments were easily differentiated from noise in the density maps. After separately determining the protein-ligand structures, the structures of the protein-ligand complexes were determined using fragment cocktails consisting of four different ligands. Therefore, the use of compound cocktails for fragment screening with cryo-EM is an efficient and timesaving method for increasing throughput.

### 4.5 Antibody design

Antibodies have been used as therapeutic tools using various approaches, such as blocking the binding interface and inhibiting activity (Carter and Rajpal, 2022). In particular, antibody–drug conjugates and antibody-based proteolysis-targeting chimeras (AbTACs) are emerging fields of antibody therapy (Fu et al., 2022; Zhao et al., 2022). A critical step in antibody-based therapeutic strategies is the acquisition of appropriate antibodies with high target specificity and affinity. The most popular approach for developing new antibodies is to screen antigen-binding complementarity-determining region (CDRs) sequences using a phage library (Alfaleh et al., 2020). Other approaches include analyses of genomic sequence information from target-responding B cells extracted from infected samples and direct peptide sequence information of antibodies in the serum using mass spectrometry (Ionov and Lee, 2022). Methodologies for computer-aided antibody design have also been developed, such as antibody–antigen docking (Hummer et al., 2022). Although diverse approaches in antibody design have accelerated development speed, structural validation of the designed antibody on-target, as expected, is still a rate-limiting process using crystallography. Owing to the lack of a crystallization step, the cryo-EM approach is advantageous for determining the structures of the target–antibody complex at high resolution. Moreover, after acquiring the initial structure of the target–antibody complex, further improvements based on structural information are easily achieved. In this subsection, we describe new techniques for supporting the design of antibodies using cryo-EM: epitope mapping and the direct determination of antibodies (Nilvebrant and Rockberg, 2018; Schardt et al., 2022).

The cryo-EM-based polyclonal epitope mapping (cryo-EMPEM) method was developed in the late 2010s for screening purposes. Cryo-EMPEM determines the sequence of polyclonal antibodies from the electron density map of the antibody-target complex, unlike previous approaches such as B cell sequence analysis or mass spectrometry analysis of polyclonal antibodies. This approach integrates antibody selection and structural determination. As a recent example of cryo-EMPEM, a novel approach was described for antibody discovery in early 2022, targeting human immunodeficiency virus-1 (HIV-1) envelope glycoprotein (Figure 4E) (Antanasijevic et al., 2022a). Serum-derived antigen-bound polyclonal antibody sequences were identified using cryo-electron microscopy density maps. For this purpose, the animals were immunized with labeled antigens to produce antigen/antibody complexes that were then analyzed using cryo-EM to identify epitopes. After reconstruction, the amino acid sequences of the antibodies were determined from density maps using a novel algorithm developed by the same research group. The main aim of this algorithm is to match density maps with the antigen-binding-specific B-cell next-generation sequencing database to rapidly obtain epitope information that can be used for the rational design of therapeutics. By the end of 2022, the same research group adapted this approach to analyze polyclonal antibody responses using whole viral particles for non-enveloped viruses with icosahedral capsids, indicating that this method is an effective way to identify antibodies and map epitopes (Antanasijevic et al., 2022b).

## 5 Examples of cryo-EM in structure-based drug design

To the best of our knowledge, none of the currently approved drugs have been designed using cryo-EM structures, although we have noted studies and ongoing efforts to design drugs/inhibitors aided by available cryo-EM structures, or using cryo-EM to solve the binding modes of newly designed drugs or target protein structures.

Recently, Garibsingh et al., 2021 designed inhibitors of alanine–serine–cysteine transporter 2 (ASCT2), a sodium-dependent neutral amino acid transporter, for SBDD. ASCT2 is responsible for amino acid homeostasis in peripheral tissues (Liu et al., 2018b). Unlike under physiological conditions, the ASCT2 protein is upregulated in various cancer types, such as leukemia, prostate cancer, and breast cancer, by the c-MYC transcription factor and increases the transport of glutamine into cells, thus stimulating proliferation (Scalise et al., 2018). Several *in vivo* studies have shown that the inhibition of ASCT2 decreases intracellular glutamine levels and, hence, tumor size (Wang et al., 2015; Ni et al., 2019). Thus, ASCT2 is a valuable pharmaceutical target. However, clinical inhibitors are still unavailable because of the lack of understanding of their pharmacological features. Garibsingh et al., 2021 combined computational modeling with cryo-EM structures of ASCT2 to design several effective inhibitors. They then selected a high-potency inhibitor, *Lc*-BPE, to solve the cryo-EM structure of the ASCT2–inhibitor complex, which showed the binding mode of the inhibitor to the protein. This study not only described the rational design of inhibitors, but also led to the design of more potent inhibitors by revealing the binding modes of inhibitor/protein complexes, showing that this combinatorial approach may be effective for designing drugs against challenging proteins in the same family.

Two nonpeptide glucagon-like peptide-1 (GLP-1) agonists, 1) orforglipron (LY-3502970) and 2) danuglipron (PF-06882961), developed by Eli Lilly and Company and Pfizer, respectively, are other examples of the contribution of cryo-EM in drug discovery. The development of these drugs was not initiated by following SBDD procedures, but the structures of GLP-1 receptor (GLP-1R)–drug complexes revealed an unknown mechanism of action for orforglipron and danuglipron (Kawai et al., 2020; Griffith et al., 2022). Activated GLP-1R, a class B GPCR, upregulates Ca^2+^-induced insulin secretion in pancreatic β cells through the GLP-1R–adenylyl cyclase–cAMP signaling axis (Zhang et al., 2017; Wen et al., 2022). Therefore, GLP-1R is a candidate of the drug target for patients with type 2 diabetes and obesity. Although small-molecule agonists are available to treat obesity and diabetes, there is still a desire to design and develop small molecules as an oral therapy that makes the treatment easier (Donnelly, 2012). Initial studies have shown that orforglipron effectively lowers glucose levels in both the humanized GLP1-R transgenic mice and the non-human primates (Kawai et al., 2020). The high-resolution structure (≈3 Å) of GLP-1R with orforglipron using cryo-EM revealed a unique binding pocket and showed the binding mechanism of the compound. Danuglipron was discovered using high-throughput screening, and its structure in the GLP-1R complex was determined using cryo-EM at 2.5 Å resolution (Griffith et al., 2022). Both drugs interacted with human/primate-specific W33 residues in the extracellular domain of GLP-1R, but not in the GLP-1-binding domain, and activated the GLP-1R downstream signals. The binding modes of the two drugs were not identical (Wan et al., 2023); however, both stabilized extracellular domain and formed structures similar to the active form of the GLP-1-GLP-1R complex. In a recent report after phase 2 clinical studies (ClinicalTrials.gov ID: NCT05048719 and NCT05051579), orforglipron showed pharmacodynamic and safety profiles similar to those of already approved injectable drugs and promising results for phase 3 studies (Frias et al., 2023; Wharton et al., 2023). Similarly, phase 2 studies (ClinicalTrials.gov ID: NCT03985293) on danuglipron for the treatment of patients with type 2 diabetes have shown promising results (Saxena et al., 2023) and phase 2 studies on obesity treatment are still ongoing (ClinicalTrials.gov ID: NCT04707313). Although the drug compounds have not been designed using SBDD, the binding modes of both orforglipron and danuglipron have been shown using a cryo-EM structure, which may lead to the design of more effective GLP-1R agonists in the future.

Another case is immunomodulatory drugs (IMiDs) and cereblon (CRBN) E3 ligase modulatory drugs (CELMoDs). Thalidomide was reported as an effective drug for erythema nodosum leprosum patients in 1965, and received FDA approval in 1998 (Bartlett et al., 2004). After reported as an inhibitor of TNF-α production (Moreira et al., 1993), it was discovered that thalidomide and its analogues including lenalidomide and pomalidomide, functioned as the immunomodulator (Davies et al., 2001). In 2010, CRBN was revealed as a direct target of thalidomide (Ito et al., 2010), and in 2014, complex structures of CRBN and damage specific DNA binding protein 1 (DDB1) with IMiDs (thalidomide and its analogues) were identified (Fischer et al., 2014). Subsequently, the potency of IMiDs was reported as a degrader by connecting between CRBN–DDB1 and neosubstrates, which were named because it is not original substrates but IMiDs-mediated new substrates (Gandhi et al., 2014; Kronke et al., 2014; Lu et al., 2014). In 2016, the mechanism of selectivity of CRBN–DDB1 to neosubstrates was unveiled in the structural aspects using X-ray crystallography (Petzold et al., 2016). In the same year, CC-885 was identified as a new degrader targeting GSPT1 with a GSPT1–CC-885–CRBN–DDB1 complex structure using X-ray crystallography (Matyskiela et al., 2016). The updated information of structures provided insights to design a novel or optimized degrader. In 2022, the binding modes of two CELMoDs CC-92480 in recruiting phase I/II (mezigdomide, ClinicalTrials.gov ID: NCT03989414) and CC-220 in recruiting phase II (iberdomide, ClinicalTrials.gov ID: NCT05199311), the enhanced version of IMiDs, to CRBN–DDB1 were unveiled using cryo-EM as well as structures of CRBN–DDB1 apo and complex forms with pomalidomide (Watson et al., 2022). By using cryo-EM, a sensor loop structure in the open state of CRBN was newly identified, and the neosubstrate-recruiting process was also inferred. The CRBN–DDB1 complex structures with IMiDs and CELMoDs aided to design novel or more efficient degraders with lowered toxicity targeting disease-related molecules, as well as understand the mechanism as modulators and degraders.

In addition to the drug development, structure of antibody-antigen complex is also applicable in the vaccine developement (Kwong et al., 2020). The process is opposite to the structure-based antibody design. Recently, Pfizer rationally designed an antigen against the respiratory syncytial virus (RSV) for vaccine development (Che et al., 2023). The RSV is a life-threatening virus that causes severe bronchiolitis and pneumonia in infants and older adults. Currently, no vaccine is available, although efforts have been ongoing for almost 60 years (Mazur et al., 2018). The key antigen in vaccine research is the RSV fusion (F) glycoprotein, which plays a role in the fusion of the viral and host cell membranes during cell entry. The metastable pre-fusion conformation of RSV F has been proposed as a target for potent neutralizing antibodies (Ngwuta et al., 2015). Therefore, the Pfizer team generated a stabilized version of the prefusion conformation of the RSV F antigen for vaccine development, based on the following hypothesis: neutralizing antibodies are elicited more effectively if the stability of the prefusion conformation is high under stress conditions. They engineered the ectodomain of RSV F and generated almost 400 constructs using the crystal structure of the RSV F protein in complex with D25, a pre-fusion-specific antibody. After analyzing the stability and immunogenicity of the engineered constructs, the most potent stabilized pre-fusion RSV F (847A) was selected, and the crystal structure of 847A alone was solved to confirm its pre-fusion structure. The cryo-EM structure of 847A was also reconstructed using two different pre-fusion conformation-specific Fabs (AM14 and AM22), confirming the integrity of the pre-fusion epitopes. The RSV vaccine candidate is currently undergoing clinical trials (ClinicalTrials.gov ID: NCT04424316 and NCT05035212) with promising preliminary results (Kampmann et al., 2023; Walsh et al., 2023). This study shows that cryo-EM can not only be used in the first step of SBDD, but is also useful for determining engineered drug/target protein structures, helping researchers understand binding modes and/or confirm structural characteristics.

## 6 Conclusion and future perspectives

SBDD has emerged as the most common and effective approach for designing therapeutics and optimizing potent and efficient drugs. Initially, SBDD relied heavily on crystal structures because of the low resolution of cryo-EM structures. However, recently, advanced techniques in cryo-EM have led to high-resolution determination of the structures of various membrane proteins and drug targets that were previously inaccessible using other biophysical methods. The increasing number of cryo-EM structures of drug-target proteins and their near-atomic resolution is expected to drive the popularity of this approach in SBDD. This review highlights the considerable potential of cryo-EM in drug development, with the atomic resolution of cryo-EM structures providing crucial insights into ligand–target interactions and activation/inhibition mechanisms of drug-target proteins. Moreover, ongoing developments in specialized techniques required for cryo-EM-based drug discovery are continually enhancing its applicability and efficiency. Although many current SBDD studies use both cryo-EM and X-ray crystallography, cryo-EM is expected to lead SBDD efforts within the next few years, producing innovative and highly effective therapeutics. The capacity of cryo-EM structures to provide atomic-level details of drug–target interactions and activation mechanisms makes them powerful tools for drug discovery. As cryo-EM technology continues to evolve and the number of high-resolution structures increases, the impact of this method on drug development is expected to increase considerably. In conclusion, the combination of cryo-EM structures at atomic resolution and newly developed techniques makes cryo-EM an invaluable tool for SBDD. With its potential to reveal intricate details of drug–target interactions and activation mechanisms, cryo-EM is poised to become the leading method for designing innovative and potent therapeutics in the near future.

## Figures and Tables

**Table:** 

**3D-QSAR**	three-dimensional quantitative structure-activity relationship
**3DVA**	3D variability analysis
**5-MTHF**	5-methyltetrahydrofolate
**AbTAC**	antibody-based proteolysis-targeting chimera
**ACST2**	alanine–serine–cysteine transporter 2
**AWI**	air-water interface
**BRIL**	apocytochrome b_562_RIL
**CAK**	cyclin-dependent kinase-activating kinase
**CC**	cross-correlation
**CCD**	charge-coupled device
**CDK**	cyclin-dependent kinase
**CDR**	complementarity-determining region
**CELMoDs**	cereblon E3 ligase modulatory drugs
**CFEG**	cold field-emission gun
**CMOS**	complementary metal oxide semiconductor
**CRBN**	cereblon
**cryo-EM**	cryogenic electron microscopy
**cryo-EMPEM**	cryo-EM-based polyclonal epitope mapping
**cryo-ET**	cryo-electron tomography
**cryo-TEM**	cryogenic transmission electron microcopy
**CTF**	contrast transfer function
**CTR**	calcitonin receptor
**DARPin**	designed ankyrin repeat protein
**DDB1**	damage specific DNA binding protein 1
**DED**	direct electron detector
**DLS**	dynamic light scattering
***E. coli***	*Escherichia coli*
**EM**	electron microscopy
**EMDB**	Electron Microscopy Data Bank
**EMPIAR**	Electron Microscopy Public Image Archive
**ER**	endoplasmic reticulum
**Fab**	antigen-binding fragment
**FBDD**	fragment-based drug design
**FDA**	the United States Food and Drug Administration
**FEG**	field-emission gun
**FSC**	Fourier shell correlation
**GABA**_**A**_	γ-aminobutyric acid receptor subtype-A
**GDH**	glutamate dehydrogenase
**GFP**	green fluorescent protein
**GLP-1**	glucagon-like peptide-1
**GLP-1R**	glucagon-like peptide-1 receptor
**GPCR**	G protein-coupled receptor
**HHAT**	human Hedgehog acyltransferase
**HIV-1**	human immunodeficiency virus-1
**IMiDs**	immunomodulatory drugs
**KDELR2**	ER lumen protein-retaining receptor 2
**κOR-ICL3**	kappa opioid receptor
**MAPS**	monolithic active pixel sensor
**MBP**	maltose-binding protein
**MCU**	mitochondrial calcium uniporter
**microED**	microcrystal electron diffraction
**Ni-NTA**	nickel–nitrilotriacetic acid
**NMR**	nuclear magnetic resonance
**NTCP**	sodium/bile acid cotransporter
**PDB**	Protein Data Bank
**PGS**	glycogen synthase domain of *Pyrococcus abyssi*
**PKM2**	pyruvate kinase M2
**PrAC**	protein A domain C
**PrAD**	protein A domain D
**PrG**	protein G
**RBD**	receptor-binding domain
**RSV**	respiratory syncytial virus
**RSV F**	respiratory syncytial virus fusion glycoprotein
**SBDD**	structure-based drug design
**SPA**	single-particle analysis
**TEM**	transmission electron microscopy
**UCP1**	uncoupling protein 1
**VPP**	Volta phase plate
**XFEL**	X-ray free electron laser
